# Effect of low-molecular-weight heparin in women undergoing frozen-thawed embryo transfer cycles: a retrospective cohort study

**DOI:** 10.1186/s12884-023-05634-1

**Published:** 2023-05-10

**Authors:** Bo Sun, Lu Li, Xiaoli Chen, Yingpu Sun

**Affiliations:** 1 Center for Reproductive Medicine, The First Affiliated Hospital of Zhengzhou University, No.1, Jianshe East Road, 450052 Zhengzhou, Henan Province China; 2 Henan Key Laboratory of Reproduction and Genetics, The First Affiliated Hospital of Zhengzhou University, No.1, Jianshe East Road, 450052 Zhengzhou, Henan Province China

**Keywords:** Low molecular weight heparin, FET, Recurrent pregnancy loss, Recurrent implantation failure, IVF

## Abstract

**Background:**

Recurrent pregnancy loss (RPL) and recurrent implantation failure (RIF) during in vitro fertilization (IVF) treatment are still tough problems without effective treatments; thus, they are important research topics. There is controversy on whether low molecular weight heparin (LMWH) improves pregnancy outcomes in women with unexplained RPL and RIF. Moreover, currently, there is a paucity of reports on the role of LMWH in the entire population undergoing frozen-thawed embryo transfer (FET) cycles. This study aimed to estimate the effects of LMWH on pregnancy outcomes in women undergoing FET cycles.

**Methods:**

There were 1881 female patients included in the study. Of the 1881 patients, 107 underwent preimplantation genetic diagnosis cycles, which were analyzed individually. The patients were divided into two groups: the LMWH group received injections of 4100 IU/d LMWH from the day of transfer until 14 ± 2 days posttransplant, the control group was the comparison group (without LMWH use). The baseline characteristics and reproductive outcomes of the patients were reviewed.

**Results:**

Of the 1774 women with normal FET cycles, no significant differences were found in the number of embryos implanted (1.31 ± 0.02 vs. 1.28 ± 0.02), embryo implantation rate, biochemical pregnancy rate, clinical pregnancy rate, live birth rate, late abortion rate, and ectopic pregnancy rate between the two groups. The LMWH group had a higher early abortion (17.8% [76/427] vs. 12.5% [55/439], p = 0.030). In the sub-group analysis, among the patients who underwent more than four transfers, the LMWH group had a lower late abortion rate (1.7% [1/60] vs. 13.2% [7/53], p = 0.043). Similarly, of the 107 women who underwent preimplantation genetic diagnosis cycles, the reproductive outcomes were comparable between the two groups.

**Conclusion:**

In the general population and PGD patients, LMWH did not improve pregnancy outcomes. Therefore, the routine use of LMWH is not recommended for early treatment.

**Supplementary Information:**

The online version contains supplementary material available at 10.1186/s12884-023-05634-1.

## Background

For those infertility couple, in vitro fertilization (IVF)/intracytoplasmic sperm injection (ICSI) is one of the most effective and successful assisted reproductive technologies (ARTs). Infertility affects approximately 15% of couples, and IVF/ICSI contributes to 1–5% of all newborns in developed countries. Embryo implantation, a low-efficiency process in the menstrual cycle and assisted reproductive technologies, is a key step in establishing pregnancy [1, 2]. Therefore, it is imperative to identify effective treatments. Meanwhile, recurrent pregnancy loss (RPL) and recurrent implantation failure (RIF) during IVF treatment are still tough issues without effective treatments [3, 4]; thus, they are hot research topics.

RPL is characterized by the occurrence of two or more pregnancy failures before 20–24 weeks of gestation, which affect approximately 2.5% of couples of childbearing age [5–7]. RPL can be caused by chromosomal abnormality, infection, structural and functional abnormalities of the reproductive system, and autoimmune disorders. Although various therapies have been evolved to prevent pregnancy loss in these patients, effective treatment are still elusive and urgently needed. Current studies have demonstrated that low molecular weight heparin (LMWH) has the effect in improving reproductive outcomes in unexplained RPL; however, the results are conflicting [8–10].

There is no standardized definition of RIF. Nonetheless, RIF is defined as three or more consecutive transfers of at least four high-quality embryos in fresh or frozen cycles without clinical pregnancy in most studies [11, 12]. RIF can be caused by chromosomal abnormalities, uterine anatomical abnormalities, and maternal immune dysfunction [13]. Previous studies have estimated the function of LMWH in RIF, but the conclusions are controversial [14–16].

Heparin was discovered in 1916, late in the 1930s, unfractionated heparin (UFH), the first therapeutic form was introduced. Currently, a variety of different types of heparin are clinically applied, include UFH, LMWH, and synthetic heparins [17]. LMWH has a longer half-life, more stable dose-response relationship, better safety profile, reduced monitoring requirement, shorter oligosaccharide/monosaccharide chain, and higher anti-Xa/anti-IIa ratios, which make it more attractive than other heparin forms [18]. Since the anticoagulative and anti-inflammatory function of LMWH, it is now extensively used for the treatment of RPL and RIF, either alone or in combination with other agents. However, it tends to be broadly used in frozen-thawed embryo transfer (FET) cycles in IVF/ICSI treatment. Our study aimed to investigate the effect of LMWH on pregnancy outcomes in women with different numbers of transfer cycles, ages, numbers of transferred embryos, and preimplantation genetic diagnosis (PGD) cycles.

## Methods

### Study design and patients

In total, 1881 of patients were enrolled in this study. Among them, 107 underwent PGD cycles, which were analyzed individually. All the data were from Clinical Reproductive Medicine Management System/Electronic Medical Record Cohort Database (CCRM/EMRCD). The inclusion criteria were as follows: (1) underwent FET cycles; (2) had at least one failure of embryo transfer (including fresh embryo transfer or FET cycles). The exclusion criteria were as follows: (1) endometriosis and/or adenomyosis; (2) uterine malformation, including congenital uterine dysplasia, uterine fibroids, endometrial polyps, and intrauterine adhesions; (3) tubal factors, including hydrosalpinx; (4) LMWH contraindications, such as active bleeding; and (5) other autoimmune diseases, such as thyroid disorders.

### Grouping method

The patients were split into two groups depending on the use or nonuse of LMWH. The LMWH group received injections of 4100 IU/d LMWH from the day of transfer until 14 ± 2 days posttransplant. The control group was the comparison group (without LMWH use). Human β-chorionic gonadotropin (HCG) levels were measured at 14 ± 2 days posttransplant in all groups. Laboratory data included routine blood, liver function, and blood coagulation test data in the LMWH group at 14 days posttransplant. If serum HCG was positive, the injection of LMWH was continued until 35 ± 2 days posttransplant. Ultrasonography to determine clinical pregnancy 35 days after transplantation. Laboratory data, including routine blood, liver function, and blood coagulation test data, were also obtained in the LMWH group at 35 ± 2 days posttransplant to evaluate the safety of LMWH.

### Endometrial preparation

The detailed endometrial preparation protocol for freeze-thaw cycles has been described in previous article, including the classification of endometrial types and thickness measurement methods [19]. For estrogen-progesterone (EP) cycles, oral estradiol ([Progynova]; Bayer, Germany) administration began on day 2–3 of the target cycle and lasted about two weeks. When the thickness of the endometrium reaches 8 mm and above, the patient is asked to add oil-based progesterone (60 mg), at the same day, the thickness of endometrial was recorded using transvaginal ultrasound examination. To avoid cavity fluid and other unfavorable conditions, patients were hospitalized and re-measurement of endometrial thickness on the morning of the transplantation day. Luteal supplement was altered to vaginal progesterone gel (90 mg, Crinone 8%; Merck Serono) and oral dydrogesterone (20 mg Duphaston; Abbott) after embryo implantation.

### Statistical methods

IBM SPSS, 21.0 (IBM Corp., Armonk, N.Y., USA) was employed. Numerical data were shown as the mean ± standard deviation (SD), while categorical variables were shown as % (n/N). The Man-Whitney test and chi-square test were utilized for continuous and categorical variables, respectively. Two-tailed P < 0.05 was considered as statistical significance.

## Results

### Baseline characteristics and reproductive outcomes in women undergoing FET cycles

Of the 1881 patients who began FET treatment between 2020 and 2021, 107 women underwent PGD cycles. First, we analyzed 1774 women with normal FET cycles. There were 882 (49.7%) and 892 (50.3%) patients in LMWH and control groups, respectively (Table 1). The results were comparable between two groups in age (32.28 ± 0.17 vs. 32.37 ± 0.16), years of infertility (4.54 ± 0.12 vs. 4.65 ± 0.12), body mass index (BMI; 23.74 ± 0.19 vs. 23.56 ± 0.12), basal serum FSH (6.65 ± 0.10 vs. 6.60 ± 0.08), basal serum LH (7.46 ± 0.33 vs. 8.06 ± 0.38), basal serum E2 (242.40 ± 27.51 vs. 263.92 ± 33.65), AMH (4.01 ± 0.12 vs. 4.13 ± 0.12), and AFC (14.65 ± 0.23 vs. 15.21 ± 0.23) between the two groups. Also, no great differences were identified in the number of embryos implanted (1.31 ± 0.02 vs. 1.28 ± 0.02), embryo implantation rate (44.9% [519/1157] vs. 45.5% [522/1146]), biochemical pregnancy rate (52.3% [461/882] vs. 51.6% [460/892]), clinical pregnancy rate (48.4% [427/882] vs. 49.2% [439/892]), live birth rate (37.9% [334/882] vs. 39.9% [356/892]), late abortion rate (2.6% [11/427] vs. 5.0% [22/439]), and ectopic pregnancy rate (1.4% [6/427] vs. 1.4% [6/439]) between the two groups. Compared to the control group, the LMWH group had a higher early abortion rate (17.8% [76/427] vs. 12.5% [55/439], p = 0.030).

**Table 1:** Baseline characteristics and pregnant outcome of patients undergoing FET cycles

	LMWH	CONTROL	P Value
Cycle number	882	892	
Female age	32.28 ± 0.17	32.37 ± 0.16	0.644
Type of infertility			0.128
Primary infertility	315	288	
Secondary infertility	567	604	
Years of infertility	4.54 ± 0.12	4.65 ± 0.12	0.753
BMI	23.74 ± 0.19	23.56 ± 0.12	0.615
Baseline hormone levels			
FSH (mIU/mL)	6.65 ± 0.10	6.60 ± 0.08	0.573
E2 (pg/mL)	242.40 ± 27.51	263.92 ± 33.65	0.813
LH (mIU/mL)	7.46 ± 0.33	8.06 ± 0.38	0.081
AMH (ng/mL)	4.01 ± 0.12	4.13 ± 0.12	0.336
AFC	14.65 ± 0.23	15.21 ± 0.23	0.088
No. of embryo implanted	1.31 ± 0.02	1.28 ± 0.02	0.213
Embryo stage			0.285
D3	339	365	
D5	543	527	
Embryo implantation rate	44.9%(519/1157)	45.5%(522/1146)	0.739
Biochemical pregnancy rate	52.3%(461/882)	51.6%(460/892)	0.769
Clinical pregnancy rate	48.4%(427/882)	49.2%(439/892)	0.735
Live birth rate	37.9%(334/882)	39.9%(356/892)	0.378
Early abortion rate	17.8%(76/427)	12.5%(55/439)	0.030
Late abortion rate	2.6%(11/427)	5.0%(22/439)	0.061
Ectopic pregnancy rate	1.4%(6/427)	1.4%(6/439)	0.961

Note: Numbers are mean ± standard deviation; BMI = body mass index; FSH = follicle-stimulating hormone; E2 = estradiol; LH = luteinizing hormone; AMH = Anti-mullerian hormone; AFC = Antra follicular count

### Baseline characteristics and reproductive outcomes in women with different numbers of transfer cycles

To assess the effect of LMWH in different numbers of transfer cycles, we grouped the 1774 women into four groups (Table 2). There were 233 (41.7%) and 326 (58.3%) patients who underwent one transfer in the LMWH and control groups, respectively. No statistical differences were found in age, years of infertility, BMI, basal serum FSH, basal serum E2, AMH, and AFC between the two groups. Also, the data were comparable between the two group in the number of embryos implanted, embryo implantation rate, biochemical pregnancy rate, clinical pregnancy rate, live birth rate, late abortion rate, or ectopic pregnancy rates. The LMWH group had lower basal serum LH levels (6.29 ± 0.59 vs. 7.77 ± 0.64, p = 0.017) and a higher early abortion rate (17.5% [18/103] vs. 8.2% [12/147], p = 0.026) than the control group. There were 328 (51.6%) and 308 (48.4%) patients who underwent two transfers in the LMWH and control groups, respectively. Further, 181 (52.9%) in the LMWH group and 161 (47.1%) patients in the control group underwent three transfers. All baseline characteristics and pregnancy outcomes between the two groups were comparable. There were 140 (59.1%) and 97 (40.9%) patients who underwent more than four transfers in the LMWH and control groups, respectively. Patients using LMWH had fewer years of infertility (6.36 ± 0.32 vs. 7.22 ± 0.37, p = 0.041) and lower embryo implantation (40.6% [73/180] vs. 55.1% [65/118], p = 0.014) and late abortion rates (1.7% [1/60] vs. 13.2% [7/53], p = 0.043).

**Table 2:** Baseline characteristics and pregnant outcome of patients in different number of transfer cycles

No. of cycle	1			2			3			≥ 4		
	LMWH	CONTROL	P Value	LMWH	CONTROL	P Value	LMWH	CONTROL	P Value	LMWH	CONTROL	P Value
Cycle number	233	326		328	308		181	161		140	97	
Female age	32.07 ± 0.35	32.43 ± 0.28	0.392	31.43 ± 0.28	31.98 ± 0.27	0.097	32.89 ± 0.35	32.42 ± 0.39	0.427	33.79 ± 0.39	33.32 ± 0.49	0.345
Type of infertility			0.813			0.014			0.032			0.107
Primary infertility	107	153		134	97		49	28		25	10	
Secondary infertility	126	173		194	211		132	133		115	87	
Years of infertility	3.60 ± 0.19	3.93 ± 0.19	0.683	4.12 ± 0.18	4.06 ± 0.17	0.880	5.13 ± 0.27	5.73 ± 0.28	0.056	6.36 ± 0.32	7.22 ± 0.37	0.041
BMI	23.49 ± 0.22	23.15 ± 0.22	0.251	23.68 ± 0.44	23.72 ± 0.20	0.167	24.08 ± 0.25	23.79 ± 0.27	0.359	23.88 ± 0.27	24.03 ± 0.32	0.686
Baseline hormone levels												
FSH (mIU/mL)	6.78 ± 0.20	6.71 ± 0.13	0.591	6.56 ± 0.14	6.56 ± 0.12	0.613	6.56 ± 0.22	6.47 ± 0.20	0.708	6.75 ± 0.27	6.55 ± 0.27	0.854
E2 (pg/mL)	296.30 ± 47.87	286.06 ± 46.85	0.903	258.97 ± 51.71	262.11 ± 69.42	0.498	130.76 ± 26.51	264.07 ± 77.31	0.928	258.17 ± 86.04	195.07 ± 78.42	0.390
LH (mIU/mL)	6.29 ± 0.59	7.77 ± 0.64	0.017	8.65 ± 0.66	8.16 ± 0.58	0.499	6.23 ± 0.46	8.96 ± 1.09	0.072	8.21 ± 0.84	7.25 ± 0.98	0.181
AMH (ng/mL)	3.37 ± 0.20	3.60 ± 0.16	0.152	4.34 ± 0.20	4.56 ± 0.23	0.587	4.05 ± 0.26	4.41 ± 0.27	0.149	4.26 ± 0.31	4.06 ± 0.37	0.362
AFC	13.37 ± 0.43	14.17 ± 0.38	0.207	15.16 ± 0.39	15.81 ± 0.39	0.191	14.90 ± 0.51	16.11 ± 0.53	0.101	15.23 ± 0.60	15.30 ± 0.66	0.922
No. of embryo implanted	1.29 ± 0.03	1.29 ± 0.03	0.984	1.32 ± 0.03	1.30 ± 0.03	0.621	1.35 ± 0.04	1.29 ± 0.04	0.217	1.29 ± 0.04	1.22 ± 0.04	0.232
Embryo implantation rate	40.7%(122/300)	41.2%(173/420)	0.888	48.5%(210/433)	47.1%(189/401)	0.693	46.7%(114/244)	45.9%(95/207)	0.861	40.6%(73/180)	55.1%(65/118)	0.014
Biochemical pregnancy rate	46.8%(109/233)	46.9%(153/326)	0.972	56.7%(186/328)	53.2%(164/308)	0.381	55.2%(100/181)	53.4%(86/161)	0.734	47.1%(66/140)	58.8%(57/97)	0.078
Clinical pregnancy rate	44.2%(103/233)	45.1%(147/326)	0.835	52.7%(173/328)	51.3%(158/308)	0.715	50.3%(91/181)	50.3%(81/161)	0.995	42.9%(60/140)	54.6%(53/97)	0.074
Live birth rate	35.2%(82/233)	39.0%(127/326)	0.364	41.2%(135/328)	41.6%(128/308)	0.918	38.3%(70/183)	40.2%(66/164)	0.704	33.6%(47/140)	38.1%(37/97)	0.469
Early abortion rate	17.5%(18/103)	8.2%(12/147)	0.026	16.2%(28/173)	14.6%(23/158)	0.682	19.8%(18/91)	13.6%(11/81)	0.278	20.0%(12/60)	17.0%(9/53)	0.681
Late abortion rate	1.0%(1/103)	4.8%(7/147)	0.190	4.0%(7/173)	1.9%(3/158)	0.413	2.2%(2/91)	6.2%(5/81)	0.352	1.7%(1/60)	13.2%(7/53)	0.043
Ectopic pregnancy rate	1.9%(2/103)	0.7%(1/147)	0.755	1.7%(3/173)	2.5%(4/158)	0.903	1.1%(1/91)	1.2%(1/81)	1.000	\	\	

Note: Numbers are mean ± standard deviation; BMI = body mass index; FSH = follicle-stimulating hormone; E2 = estradiol; LH = luteinizing hormone; AMH = Anti-mullerian hormone; AFC = Antra follicular count

### Baseline characteristics and reproductive outcomes in different age groups

To assess the effect of LMWH at different ages, we grouped the 1774 women into four groups: ages < 30, 30–35, 35–40, and ≥ 40 years (Table 3). In the LMWH and control groups respectively, there were 260 (49.9%) and 261 (50.1%) patients aged < 30 years, 379 (51.3%) and 360 (48.7%) patients aged 30–35 years, 157 (45%) and 192 (55%) patients aged 35–40 years, and 86 (52.1%) and 79 (47.9%) patients aged ≥ 40 years. All baseline characteristics and pregnancy outcomes were comparable among the four age groups. To further specify the effect of LMWH at different ages and numbers of transfer cycles, we grouped the patients according to the number of transfer cycles in the four age groups (Supplementary 1). The LMWH group had a lower biochemical pregnancy rate (50% [13/26] vs. 78.3% [18/23], p = 0.041) and clinical pregnancy rate (38.5% [10/26] vs. 69.6% [16/23], p = 0.029) than the control group among patients aged < 30 years who underwent more than four transfers (Supplementary 1). The LMWH group had a lower late abortion rate (0.0% [0/32] vs. 19.0% [4/21], p = 0.042) among 30–35-year-old patients who underwent more than four transfers (Supplementary 1).

**Table 3:** Baseline characteristics and pregnant outcome of patients in different age

Age	< 30 years old			30–35 years old			35–40 years old			≥ 40 years old		
	LMWH	CONTROL	P Value	LMWH	CONTROL	P Value	LMWH	CONTROL	P Value	LMWH	CONTROL	P Value
Cycle number	260	261		379	360		157	192		86	79	
Female age	26.68 ± 0.14	26.85 ± 0.13	0.432	31.97 ± 0.07	31.87 ± 0.07	0.311	36.90 ± 0.11	36.97 ± 0.10	0.644	42.09 ± 0.21	41.71 ± 0.19	0.250
Type of infertility			0.039			0.308			0.499			0.038
Primary infertility	151	128		141	121		19	28		4	11	
Secondary infertility	109	133		238	239		138	164		82	68	
Years of infertility	3.28 ± 0.13	3.40 ± 0.14	0.770	4.51 ± 0.14	4.51 ± 0.17	0.400	5.57 ± 0.34	6.28 ± 0.31	0.097	6.62 ± 0.60	5.49 ± 0.49	0.366
BMI	23.27 ± 0.21	23.58 ± 0.21	0.371	23.50 ± 0.17	23.32 ± 0.18	0.441	23.87 ± 0.24	23.60 ± 0.21	0.416	26.03 ± 1.55	24.49 ± 0.54	0.231
Baseline hormone levels												
FSH (mIU/mL)	6.11 ± 0.11	6.03 ± 0.11	0.407	6.27 ± 0.12	6.50 ± 0.12	0.031	7.00 ± 0.23	6.92 ± 0.19	0.468	9.29 ± 0.58	8.10 ± 0.31	0.355
E2 (pg/mL)	310.14 ± 63.17	327.14 ± 72.27	0.866	200.06 ± 37.82	256.15 ± 57.09	0.872	234.60 ± 57.45	233.46 ± 53.64	0.557	238.39 ± 65.75	164.54 ± 52.20	0.541
LH (mIU/mL)	8.10 ± 0.68	7.91 ± 0.67	0.549	7.20 ± 0.50	8.75 ± 0.67	0.008	6.69 ± 0.63	6.75 ± 0.60	0.808	8.11 ± 1.20	8.90 ± 1.46	0.939
AMH (ng/mL)	4.85 ± 0.23	5.24 ± 0.24	0.136	4.39 ± 0.19	4.56 ± 0.20	0.315	2.93 ± 0.20	2.72 ± 0.14	0.416	1.79 ± 0.18	1.91 ± 0.18	0.525
AFC	17.13 ± 0.40	18.07 ± 0.37	0.054	15.55 ± 0.33	16.24 ± 0.33	0.158	12.11 ± 0.54	11.76 ± 0.46	0.674	7.80 ± 0.59	9.46 ± 0.65	0.042
No. of embryo implanted	1.35 ± 0.03	1.32 ± 0.03	0.482	1.32 ± 0.02	1.29 ± 0.02	0.367	1.24 ± 0.03	1.23 ± 0.03	0.778	1.29 ± 0.05	1.29 ± 0.05	0.995
Embryo implantation rate	50.3%(177/352)	49.9%(172/345)	0.910	46.9%(234/499)	48.2%(223/463)	0.693	45.6%(89/195)	42.4%(100/236)	0.496	17.1%(19/111)	26.5%(27/102)	0.097
Biochemical pregnancy rate	58.8%(153/260)	42.9%(112/261)	0.684	51.2%(194/379)	51.9%(187/360)	0.837	51.6%(81/157)	48.4%(93/192)	0.558	22.1%(19/86)	30.4%(24/79)	0.266
Clinical pregnancy rate	54.6%(142/260)	55.6%(145/261)	0.829	51.2%(194/379)	51.9%(187/360)	0.837	47.8%(75/157)	44.8%(86/192)	0.579	18.6%(16/86)	26.6%(21/79)	0.220
Live birth rate	42.7%(111/260)	47.1%(123/261)	0.309	40.6%(154/379)	41.4%(149/360)	0.835	36.3%(57/157)	35.9%(69/192)	0.943	14.0%(12/86)	19.0%(15/79)	0.383
Early abortion rate	17.6%(25/142)	11.0%(16/145)	0.112	16.0%(31/194)	12.8%(24/187)	0.383	21.3%(16/75)	10.5%(9/86)	0.058	25.0%(4/16)	28.6%(6/21)	1.000
Late abortion rate	3.5%(5/142)	2.8%(4/145)	0.975	2.6%(5/194)	5.9%(11/187)	0.108	1.3%(1/75)	8.1%(7/86)	0.105	\	\	
Ectopic pregnancy rate	0.7%(1/142)	1.4%(2/145)	1.000	2.1%(4/194)	1.6%(3/187)	1.000	1.3%(1/75)	1.2%(1/86)	1.000	\	\	

Note: Numbers are mean ± standard deviation; BMI = body mass index; FSH = follicle-stimulating hormone; E2 = estradiol; LH = luteinizing hormone; AMH = Anti-mullerian hormone; AFC = Antra follicular count

### Baseline characteristics and reproductive outcomes in different numbers of transferred embryos

To assess the effect of LMWH in different numbers of transferred embryos, we grouped the 1774 women into four groups (first group, one transferred embryo; second group, one transferred blastocyst; third group, two transferred embryos; fourth group, two transferred blastocysts) (Table 4). In the LMWH and control groups respectively, 119 (44.9%) and 146 (55.1%) patients had one transferred embryo, 488 (49.8%) and 492 (50.2%) patients had one transferred blastocyst, 220 (50.1%) and 219 (49.9%) patients had two transferred embryos, and 55 (61.1%) and 35 (38.9%) patients had two transferred blastocysts. To further specify the effect of LMWH at different ages and numbers of transferred embryos, we grouped the patients according to the number of transferred embryos at different ages (Supplementary 2). The LMWH group had a higher early abortion rate [13.0% [6/46] vs. 0.0% [0/45], p = 0.037) among patients aged < 30 years who had two transferred embryos (Supplementary 2).

**Table 4:** Baseline characteristics and pregnant outcome of patients transferred different number of embryos

No.of embryo transferred	one embryo			one blastocyst			two embryo			two blastocyst		
	LMWH	CONTROL	P Value	LMWH	CONTROL	P Value	LMWH	CONTROL	P Value	LMWH	CONTROL	P Value
Cycle number	119	146		488	492		220	219		55	35	
Female age	35.24 ± 0.52	34.23 ± 0.40	0.070	31.80 ± 0.21	32.08 ± 0.21	0.513	31.87 ± 0.35	31.97 ± 0.35	0.777	31.71 ± 0.49	31.20 ± 0.77	0.108
Type of infertility			0.841			0.299			0.662			0.611
Primary infertility	19	22		171	157		100	95		25	14	
Secondary infertility	100	124		317	335		120	124		30	21	
Years of infertility	5.67 ± 0.40	5.75 ± 0.33	0.617	4.35 ± 0.14	4.61 ± 0.16	0.692	4.42 ± 0.23	4.11 ± 0.20	0.431	4.35 ± 0.44	4.14 ± 0.48	0.990
BMI	23.99 ± 0.27	23.22 ± 0.23	0.073	23.58 ± 0.15	23.84 ± 0.17	0.450	23.39 ± 0.24	23.14 ± 0.22	0.554	26.01 ± 2.44	23.61 ± 0.59	0.741
Baseline hormone levels												
FSH (mIU/mL)	7.93 ± 0.44	7.22 ± 0.25	0.195	6.35 ± 0.10	6.36 ± 0.09	0.770	6.78 ± 0.19	6.74 ± 0.15	0.567	5.95 ± 0.23	6.46 ± 0.32	0.199
E2 (pg/mL)	217.41 ± 57.64	125.20 ± 36.38	0.477	282.16 ± 44.40	280.19 ± 51.22	0.922	197.46 ± 36.81	317.23 ± 61.79	0.819	123.42 ± 42.89	280.42 ± 209.65	0.898
LH (mIU/mL)	8.41 ± 1.03	6.87 ± 0.78	0.649	7.56 ± 0.48	8.14 ± 0.54	0.079	6.72 ± 0.54	8.19 ± 0.72	0.153	7.49 ± 0.84	11.15 ± 2.22	0.309
AMH (ng/mL)	2.53 ± 0.24	2.62 ± 0.19	0.257	4.15 ± 0.15	4.32 ± 0.16	0.513	4.22 ± 0.25	4.38 ± 0.25	0.264	5.14 ± 0.61	6.16 ± 0.94	0.599
AFC	10.91 ± 0.63	11.50 ± 0.55	0.378	15.27 ± 0.30	15.93 ± 0.29	0.124	14.92 ± 0.47	15.56 ± 0.46	0.296	16.07 ± 0.87	18.31 ± 1.29	0.169
Embryo implantation rate	30.3%(36/119)	29.5%(43/146)	0.887	54.1%(264/488)	53.9%(265/492)	0.941	39.3%(173/440)	39.0%(171/438)	0.933	41.8%(46/110)	54.3%(38/70)	0.102
Biochemical pregnancy rate	30.3%(36/119)	32.2%(47/146)	0.735	53.5%(261/488)	52.4%(258/492)	0.743	57.8%(127/220)	58.0%(127/219)	0.955	67.3%(37/55)	80%(28/35)	0.189
Clinical pregnancy rate	29.4%(35/119)	28.1%(41/146)	0.812	48.8%(238/488)	50.6%(249/492)	0.565	54.5%(120/220)	55.7%(122/219)	0.807	61.8%(34/55)	77.1%(27/35)	0.129
Live birth rate	23.5%(28/119)	20.5%(30/146)	0.559	37.1%(181/488)	40.2%(198/492)	0.311	45.5%(100/220)	48.9%(107/219)	0.475	45.5%(25/55)	60%(21/35)	0.178
Early abortion rate	20.0%(7/35)	12.2%(5/41)	0.352	20.2%(48/238)	14.5%(36/249)	0.095	11.7%(14/120)	6.6%(8/122)	0.167	20.6%(7/34)	22.2%(6/27)	0.877
Late abortion rate	0.0%(0/35)	14.6%(6/41)	0.053	2.5%(6/238)	4.4%(11/249)	0.254	3.3%(4/120)	4.1%(5/122)	1.000	2.9%(1/34)	0.0%(0/27)	1.000
Ectopic pregnancy rate	\	\		1.3%(3/238)	1.6%(4/249)	1.000	1.7%(2/120)	1.6%(2/122)	1.000	2.9%(1/34)	0.0%(0/27)	1.000

Note: Numbers are mean ± standard deviation; BMI = body mass index; FSH = follicle-stimulating hormone; E2 = estradiol; LH = luteinizing hormone; AMH = Anti-mullerian hormone; AFC = Antra follicular count

### Baseline characteristics and reproductive outcomes of patients undergoing PGD

Of the 1881 patients who began FET treatment between 2020 and 2021, 107 women who underwent PGD cycles were analyzed separately. There were 50 (46.7%) and 57 (53.3%) patients in the LMWH and control groups, respectively (Table 5). The results were comparable between two groups in age (30.00 ± 0.51 vs. 30.46 ± 0.45), years of infertility (2.42 ± 0.27 vs. 2.53 ± 0.25), BMI (23.30 ± 0.32 vs. 22.89 ± 0.33), basal serum FSH (5.98 ± 0.24 vs. 5.96 ± 0.24), basal serum LH (5.89 ± 0.74 vs. 6.98 ± 1.47), basal serum E2 (66.55 ± 10.01 vs. 71.30 ± 12.31), AMH (4.38 ± 0.45 vs. 3.81 ± 0.30), and AFC (16.62 ± 0.80 vs. 16.19 ± 0.67). No statistical differences were found in embryo implantation rate (60% [30/50] vs. 56.1% [32/57]), biochemical pregnancy rate (66% [33/50] vs. 64.9% [37/57]), clinical pregnancy rate (60% [30/50] vs. 56.1% [32/57]), live birth rate (42% [21/50] vs. 38.8% [26/57]), early abortion rate (23.3% [7/30] vs. 18.8% [6/32]), late abortion rate (3.3% [1/30] vs. 0.0% [0/32]), and ectopic pregnancy rate (3.3% [1/30] vs. 0.0% [0/32]) between the two groups.

**Table 5:** Baseline characteristics and pregnant outcome of patients undergoing PGD

	LMWH	CONTROL	P Value
Number	50	57	
Female age	30.00 ± 0.51	30.46 ± 0.45	0.467
Type of infertility			0.288
Primary infertility	12	19	
Secondary infertility	38	38	
Years of infertility	2.42 ± 0.27	2.53 ± 0.25	0.776
BMI	23.30 ± 0.32	22.89 ± 0.33	0.372
Baseline hormone levels			
FSH (mIU/mL)	5.98 ± 0.24	5.96 ± 0.24	0.955
E2 (pg/mL)	66.55 ± 10.01	71.30 ± 12.31	0.609
LH (mIU/mL)	5.89 ± 0.74	6.98 ± 1.47	0.750
AMH (ng/mL)	4.38 ± 0.45	3.81 ± 0.30	0.461
AFC	16.62 ± 0.80	16.19 ± 0.67	0.740
embryo stage			0.804
D5	31	34	
D6	19	23	
Embryo implantation rate	60%(30/50)	56.1%(32/57)	0.687
Biochemical pregnancy rate	66%(33/50)	64.9%(37/57)	0.906
Clinical pregnancy rate	60%(30/50)	56.1%(32/57)	0.687
Live birth rate	42%(21/50)	38.8%(26/57)	0.707
Early abortion rate	23.3%(7/30)	18.8%(6/32)	0.658
Late abortion rate	3.3%(1/30)	0.0%(0/32)	0.484
Ectopic pregnancy rate	3.3%(1/30)	0.0%(0/32)	0.484

Note: Numbers are mean ± standard deviation; BMI = body mass index; FSH = follicle-stimulating hormone; E2 = estradiol; LH = luteinizing hormone; AMH = Anti-mullerian hormone; AFC = Antra follicular count

## Discussion

Embryo implantation is a complicated physiological process that includes proliferation and differentiation, adhesion and migration, and extracellular matrix remodeling. It can be influenced by many factors, such as abnormal uterine cavity anatomy, reduced endometrial receptivity, immune disorders, pre-thrombotic state, advanced age, excessive BMI, abnormal thyroid function, and psychological factors. For decades, researchers have investigated effective treatments to improve pregnancy outcomes in IVF cycles.

Previous research has shown that impaired placental function may cause arterial thrombosis, which can lead to subsequent abortion. In addition, venous thromboembolism is more prevalent during gestation compared to arterial thrombosis [20]. To ensure the nutritional supply to the fetus, maternal blood flow is exchanged with the fetus through the placental intervillous space from about 10 weeks of gestation onwards [21]. In the last century, a relationship between RPL and antiphospholipid antibodies (APAs) was identified. APAs increase the generation of thrombin, leading to thrombotic damage in the placental [22]. LMWH is commonly used clinically for the treatment of acute VTE; thus, it was used to prevent miscarriage in women with APS by its antithrombotic function [23]. LMWHs may be useful in controlling endometrial differentiation and receptivity by regulating IGFBP-1, PRL, and IGF-I in assisted reproduction [24]. By increasing placental production of matrix metalloproteinases (MMPs) and tissue inhibitors metalloproteinases (TIMPs), LMWH might also regulate trophoblast invasiveness [25].

Therefore, many clinicians have attempted to use it in FET cycles to improve reproductive outcomes in ART treatment, and not just in RPL or RIF. Currently, there is little research regarding the role of LMWH in the entire population undergoing FET cycles [26]. In our study, LMWH had no obvious advantage in decreasing the risk of abortion or increasing the rate of conception in women with or without PGD. It’s reported that advanced age greatly increase the chance of adverse pregnancy outcomes, which could impair the safety of both mother and baby[27]. Dmitry et al. showed that patients aged < 35 or 35–37 years had a higher chance of a good perinatal outcome by transferring a single 5- or 3-day embryo, and patients aged > 40 years had a higher chance of a good perinatal outcome by transferring two 3-day embryos [28]. To exclude the effects of age and the number of transferred cycles and embryos, we performed further subgroup stratification analysis. Among the patients who underwent more than four transfers, the use of LMWH reduced the late abortion rate. While patients aged 30–35 years who underwent more than four transfers had a lower late abortion rate in the LMWH group. In this study, LMWH reduced late abortion when the patients underwent more than four transfers, which is consistent with the findings of studies on RPL and RIF [29]. However, previous studies have generally been insufficiently subgrouped, have observed a simple outcome indicator, and few have explored the role of LMWH on late abortion rate. Studies have shown that the main causes of late abortion were APAs, cervical incompetence, infections, and placental insufficiency [30]. All the patients in our study were APAs negative, and LMWH did not show the tendency of reducing late abortion rate in the whole population, therefore, it’s unreasonable to draw the conclusion that LMWH make contribution for the protection of late abortion. A meta-analysis also reported that LMWH could not significantly reduced the chance of abortion in non-thrombophilic patients in fresh cycles [31]. To further investigate the relationship between LMWH and late abortion, large sample and multi-center studies are needed. Genetic factors are the main causes of early miscarriages [32]. In our study, we excluded this factor from the PGD. However, LMWH has no obvious advantage in decreasing the risk of abortion or increasing the pregnancy rate. A limitation in the PGD cycles was the insufficient samples to process the subgroup analysis.

In contrast to other heparin components, LMWH has a favorable safety profile as an anticoagulant. Many studies reported the effectiveness of LMWH as a therapeutic method for unexplained RPL (URPL). However, due to the mechanism of LMWH, side effects such as allergic reactions and thrombocytopenia are inevitable in pregnant women [33]. Therefore, To avoid some possible side effects such as bleeding, rash, liver and kidney impairment, patients on LMWH should be strictly monitored [34]. Moreover, a study reported some maternal and fetal complications after using LMWH for the treatment of URPL [9]. In our study, LMWH increased early abortion rate in the whole population, further subgroup analysis showed that this happened only in patients who had embryo implantation failure once and aged under 30. However, the limited sample could not support us to draw the conclusion that using LMWH resulted higher early abortion rate. Given the side effects of LMWH and few studies explored its function on early abortion, We proposed that the using of LMWH in these younger patients caused abnormal bleeding and induced pregnancy loss in early stage. In the light of the above findings, we need to balance the use of LMWH. According to our findings, LMWH is not recommended for routine use in patients without confirmed immune disorders in the first two cycles in FET treatment.

## Conclusions

In the general population, women using LMWH had higher early abortion rate compared to the control group, subgroup analysis showed it only presented in patients who had embryo implantation failure once and aged under 30. However, LMWH did not improve the pregnant outcomes in the general population and PGD patients, therefore, the routine use of LMWH is not recommended for early treatment.

## Electronic supplementary material

Below is the link to the electronic supplementary material.

**Additional file 1:** Supplementary 1 and 2

## Data Availability

All data generated or analyzed during this study are included in this published article [and its supplementary information files].

## List of abbreviations

APAs: Antiphospholipid antibodies
ARTs: Assisted reproductive technologies
EP: Estrogen-progesterone
FET: Frozen-thawed embryo transfer
HCG: Human β-chorionic gonadotropin
IVF: In vitro fertilization
ICSI: Intracytoplasmic sperm injection
LMWH: Low molecular weight heparin
MMPs: Matrix metalloproteinases
PGD: Preimplantation genetic diagnosis
RIF: Recurrent implantation failure
RPL: Recurrent pregnancy loss
SD: Standard deviation
TIMPs: Tissue inhibitors metalloproteinases
UFH: Unfractionated heparin
